# Treatment of Acetabular Fractures by Acute Arthroplasty: A Comparison of Trabecular Metal Cages With a Cemented Bimobile Acetabular Component Versus a Polyethylene Liner

**DOI:** 10.7759/cureus.104611

**Published:** 2026-03-03

**Authors:** Melissa C Soderquist, Diana V Rijo, Marissa Pazik, Jennifer E Hagen, Richard Vlasak

**Affiliations:** 1 Department of Orthopaedic Surgery and Sports Medicine, University of Florida, Gainesville, USA

**Keywords:** acetabular fracture, dual mobility, joint replacement, total hip arthroplasty, trabecular metal cage

## Abstract

Introduction: The treatment of acetabular fractures with pelvic reconstruction is technically challenging and labor-intensive and carries high complication rates. This retrospective review compares the treatment of acetabular fractures with a trabecular metal cage with a cemented bimobile acetabular component versus a cemented polyethylene liner and dual mobility articulation, in addition to open reduction internal fixation (ORIF) of the acetabulum.

Methods: This retrospective single surgeon review included all patients aged 18 or older who presented with an acetabular fracture between 2015 and 2025 at an academic, tertiary trauma and elective arthroplasty referral center (UF Health Gainesville). The surgical technique included treatment with a trabecular metal cage with a cemented polyethylene liner or a metal bimobile acetabular component.

Results: Forty-six patients were included in the final sample, 26 in the metal bimobile component group and 20 in the polyethylene component group. Patients receiving a polyethylene component (63.3±14.9) were significantly younger than those receiving a metal bimobile component (73.1± 10.7, p=0.03). Patients receiving a polyethylene component had a shorter hospital length of stay than patients receiving a metal bimobile component (p=0.02). The rate of heterotopic ossification was similar between groups at a minimum of six months post-surgery, as assessed via Brooker classification.

Conclusions: We found comparable outcomes in the treatment of acetabular fractures with a trabecular metal cage using either a cemented bimobile component or a polyethylene liner with subsequent ORIF. Either component may serve in fixation for elderly acetabular fractures, particularly in patients with pre-existing osteoarthritis, osteoporosis, concomitant femoral head fractures, and marginal impaction.

## Introduction

Open reduction internal fixation (ORIF) for displaced acetabular fractures can provide excellent clinical outcomes [1]. Acetabular fractures in the elderly require special considerations due to age, pre-existing osteoarthritis (OA), fracture pattern, the presence of osteoporosis, and the importance of post-operative mobilization [2]. Management options can include non-operative treatment, ORIF, acute or delayed total hip arthroplasty (THA), or a combination of ORIF and THA [3]. Previous investigations have shown inferior clinical outcomes in elderly acetabular fracture patients treated either non-operatively or with ORIF [4].

The ability to allow immediate weight bearing and early mobilization is of paramount importance in the management of elderly acetabular fractures [5]. Weight bearing following ORIF alone is typically reduced for 6-8 weeks following fixation. The use of a trabecular metal cage with ORIF allows for potential bone ingrowth of the tantalum cup and allows for immediate weight bearing [4], which may reduce complications associated with prolonged immobilization [6,7] and may reduce the need for further surgery [8,9].

Acute THA in acetabular fractures is technically challenging and carries higher complication rates compared to primary THA [2]. Elderly acetabular fractures that undergo ORIF have shown inferior outcomes, and up to two-thirds of patients may subsequently require THA [10]. Additionally, delayed THA following acetabular fracture has inferior functional outcomes compared to elective THA for primary OA [11,12]. THA dislocation in the setting of an acetabular fracture with ORIF has been reported to be approximately 6.1% when performed simultaneously [6]. Dual mobility articulations can enhance the stability of these constructs, thereby preventing dislocations and reoperations. Dual-mobility cups have been used more frequently for complex instability cases due to their improved jump distance and reduced head-neck impingement in comparison to large-diameter heads or constrained implants [12,13]. When used for primary THA, dual-mobility cups have shown up to 99% survival free from cup revision for dislocation at two years [14]. In revision settings, dislocation-free survival rates at five years have been found to be 96% [13]. The senior author historically treated geriatric acetabular fractures with significant articular involvement with ORIF and a cemented polyethylene liner; when mobile bearings became readily available in the US market, a switch from cemented polyethylene to mobile bearings occurred due to presumed implant duration and improved stability.

This investigation seeks to compare clinical outcomes for a single surgeon between acetabular fractures treated with ORIF and an acute trabecular metal cage with a metal bimobile component versus a polyethylene liner. We hypothesized that patients receiving the metal bimobile component would have better outcomes compared to the polyethylene component, specifically a lower dislocation rate. 

## Materials and methods

**Figure 1:**
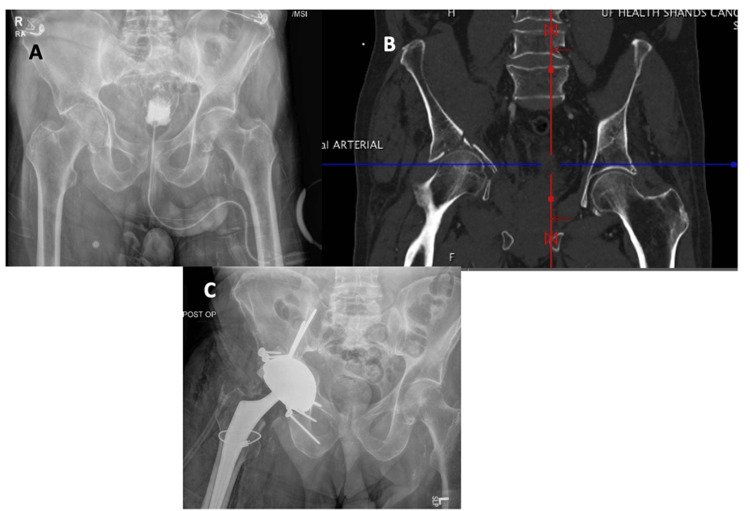
A 72-year-old man with (A) pre-operative AP pelvis showing a right acetabular fracture, (B) pre-operative CT scan showing a right acetabular fracture, and (C) post-operative AP pelvis showing ORIF with acute THA using a metal bimobile acetabular component. THA: Total hip arthroplasty; ORIF: open reduction internal fixation

This is an institutional review-board approved, retrospective cohort, single-surgeon, case series from an academic, tertiary trauma and elective arthroplasty referral center (UF Health Gainesville). All acetabular fractures in patients above 18 years of age who presented between 2015 and 2025 treated with a THA and ORIF were reviewed. Patients must have been treated with a trabecular metal cage with a cemented polyethylene liner or a metal bimobile acetabular component (Figure 1). Patients with prior ipsilateral THA were excluded. After the injury and assessment at the trauma center, the management decision was made by the orthopedic trauma and adult reconstruction teams, taking into consideration age, comorbidities, bone quality, pre-existing OA, and fracture characteristics.

Surgical approach and technique

**Figure 2:**
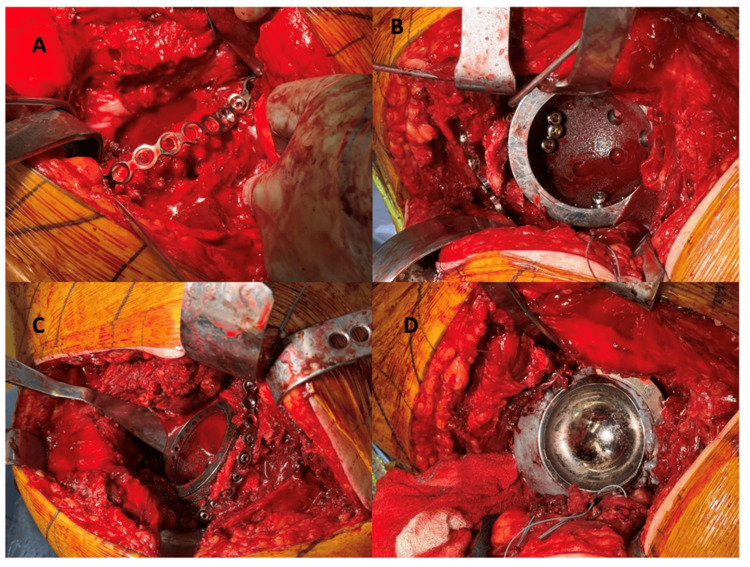
(A) 3.5mm recon plate contoured to the posterior wall and anchored to the ischium, (B) trabecular metal cage in place with three 4.5mm screws in the posterior superior quadrant and one in the posterior inferior quadrant, (C) completion of the ORIF using the plate with screws placed into the plate itself, and (D) the final acetabular construct with a trabecular metal cage, a 3.5mm plate and a cemented bimobile acetabular component. ORIF: Open reduction internal fixation

**Figure 3:**
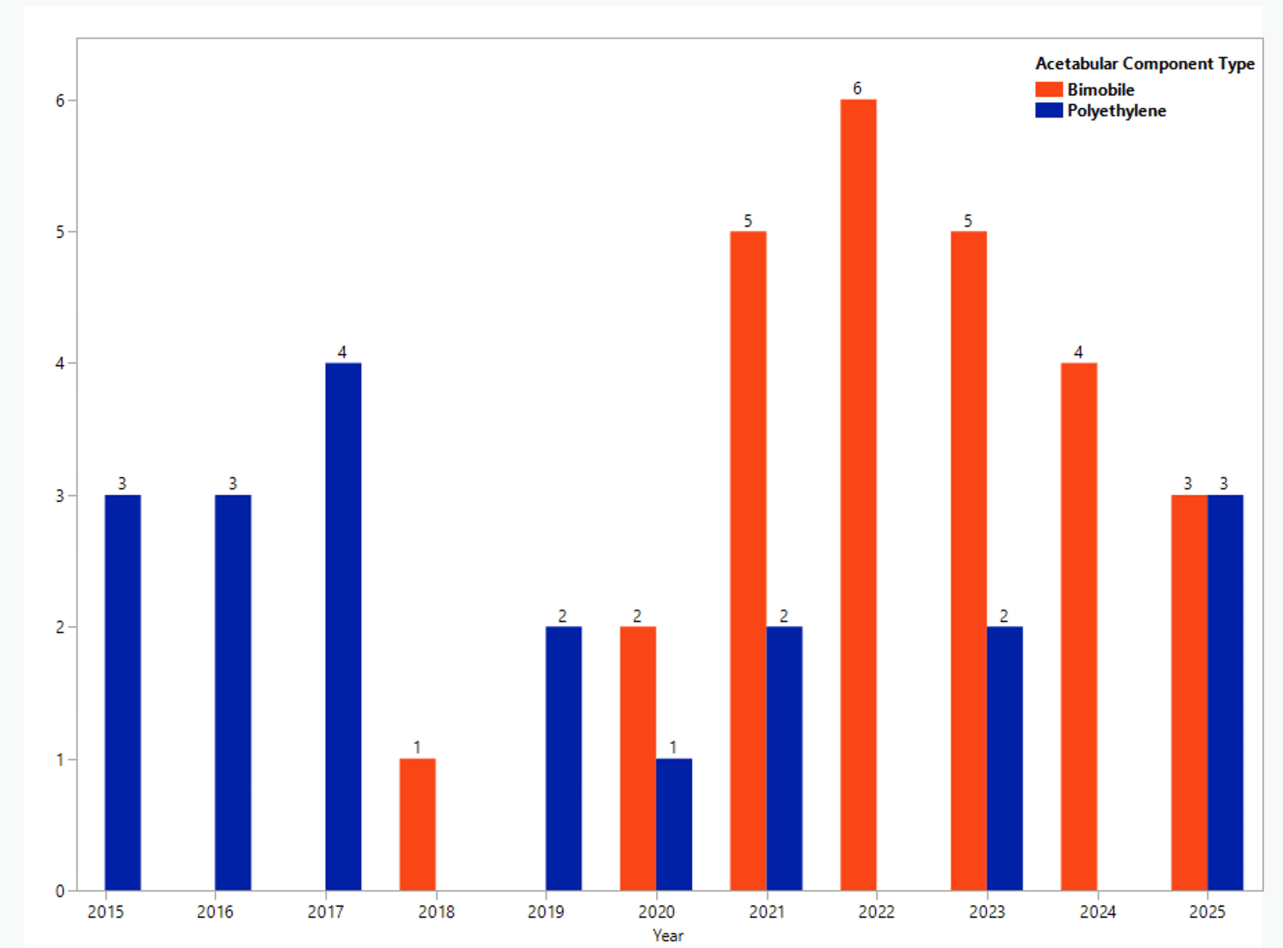
Acetabular component type by year

All patients underwent a Kocher-Langenbeck approach to the hip. A trabecular metal cage is placed and fixed using four 4.5mm screws directly into the tantalum itself. The posterior wall plate is then applied and also drilled directly into the tantalum itself for added stability. The pelvis is then tested for stability and should move as one unit. Either a cemented bimobile acetabular component or a cemented polyethylene liner is then placed. Images for each step can be found in Figure 2. The femoral component is then placed, and dual mobility articulation is reduced and tested for stability. External rotators are repaired and standard closure is performed. The selection between implant components (polyethylene or bimobile) is historical and based on market availability. As dual mobility became more widely available, the senior surgeon switched techniques and component use. Figure 3 details the number of components performed from 2015 to 2025, with the increase in metal bimobile occurring in later years.

Post-operative care

After surgery, all patients remained in the hospital and received standard perioperative antibiotics as well as antithrombotic prophylaxis. All patients had physiotherapy. After all surgical procedures, the operative surgeon determined the patient’s weight-bearing status. Patients’ discharge disposition was noted and all patients were seen at regular follow-up intervals, individually tailored to each patient’s recovery needs or complications.

Outcomes

Outcome measures included post-operative weight-bearing status, time to discharge, ambulatory device usage, infection, dislocation, revision operation, neurologic deficits, heterotopic ossification, and venous thromboembolism.

Statistical analysis

Statistical analyses were performed using JMP Pro 17 (SAS Institute Inc, Cary, NC). Categorical measures are summarized as counts and percentages. Continuous measures are summarized as means and standard deviations. Continuous variables were evaluated using the Mann-Whitney test for non-parametric data. Categorical variables were evaluated using Pearson x2 or Fisher’s exact test depending on the sample size. Significance was set at alpha = 0.05.

## Results

Patient demographics

A total of 46 patients met the inclusion/exclusion criteria. Twenty-five patients were male, and 21 patients were female. The average age at surgery was 68.8± 13.4 years, and the mean BMI was 27.9 ± 7.3. Comorbidities were comparable between groups, with the exception of hypertension (HTN); 22 patients in the metal bimobile group had HTN, and 11 patients in the polyethylene group had HTN (p=0.05). There was a significant difference in age at surgery between the two liner types, with patients who received a polyethylene component (63.3±14.9) more likely to be younger than those receiving a metal bimobile component (73.1± 10.7, p=0.03). The average follow-up time across all subjects was 553.3 ± 843.6 days. Patients receiving the polyethylene component (1067.3 ± 1139.6 days) had a longer length of follow-up compared to those receiving the metal bimobile component (202.8 ± 192.1 days, p<.001), likely due to the difference in age. Nine subjects had no follow-up data, five in the polyethylene group and four in the metal bimobile group. Comorbidities and patient characteristics can be found in Table 1.

**Table 1:** Participant characteristics BMI: Body Mass Index; ASA: American Society of Anesthesiologists Physical Status Classification System; COPD: Chronic Obstructive Pulmonary Disease; CKD: Chronic Kidney Disease; CHF: Congestive Heart Failure; CAD: Coronary Artery Disease; PVD: Peripheral Vascular Disease. p-values for continuous variables were evaluated using the Mann-Whitney test for non-parametric data. Categorical variables were evaluated using Pearson x2 or Fisher’s exact test depending on the sample size. No statistical analyses were performed when the expected value of cells was less than five.

Variables	Metal Bimobile Component	Polyethylene Component	
	n=26	n=20	p-value
Sex (Female)	9	12	0.14
Age (years)	73.1±10.7	63.3±14.9	0.03
Follow-up (days)	202.8±192.1	1067.3±1139.6	0.00
BMI	27.3±7.2	28.6±7.4	0.49
Hypertension	22	11	0.05
ASA (III)	22	15	0.46
Smoker	15	12	0.83
COPD	5	3	N/A
CKD	6	0	N/A
CHF	5	2	N/A
CAD	7	1	N/A
PVD	2	1	N/A
Osteoporosis	11	4	N/A
Rheumatoid Arthritis	0	1	N/A

Post-operative characteristics

There was a significant difference in the length of stay (LOS) between groups (p=0.02), with 61.5% (16/26) of patients in the metal bimobile component group spending greater than one week in the hospital post-operatively. Fifteen patients with a polyethylene component were discharged in less than one week compared to 10 patients with a metal bimobile component. All but one patient in the polyethylene group were made weight-bearing as tolerated (WBAT) after surgery, and 22 patients in the metal bimobile group were made WBAT after surgery. Nineteen patients in the metal bimobile group and 17 patients in the polyethylene group used a walker or cane following surgery. Six patients in the cemented bimobile group used a wheelchair following surgery. One patient in the metal bimobile component and three in the polyethylene component group did not require assistive devices. Results can be found in Table 2.

**Table 2:** Post-operative characteristics by component type

		Metal Bimobile Component	Polyethylene Component
		n=26	n=20
Time to discharge	<1 week	10	15
	>1 week	16	5
Post-operative weight-bearing status	Non-weight bearing	4	1
	Weight bearing as tolerated	22	19
Ambulatory device	None	1	3
	Cane/Walker	19	17
	Wheelchair	6	0

Complications and reoperations

There was one patient in the polyethylene group who had a superficial infection. No patients in either group had a deep infection. No patients in either group had a dislocation or required a revision operation. Three patients in the metal bimobile group and two patients in the polyethylene group had a neurologic deficit in the peroneal division of the sciatic nerve. Additionally, two patients in the metal bimobile group had a DVT versus zero in the polyethylene group.

Heterotopic ossification

Most patients in both groups were classified as Brooker 0-1 (Metal Bimobile = 20, polyethylene = 15). There was one patient in each group who was classified as Brooker 3. Only one patient had a Brooker classification of 4, and they received the metal bimobile component. We were unable to determine classification for eight patients due to no six-month follow-up or patients being deceased at six months. 

## Discussion

Acute arthroplasty for acetabular fractures in the elderly is associated with improved patient outcomes in comparison to those undergoing delayed THA, particularly when unable to achieve an anatomic reduction [10]. Anatomic reductions in these patients are able to be achieved in up to 50% of elderly acetabular fractures [15-17]. Fracture morphology and patient characteristics that can indicate a decreased likelihood of achieving an anatomic reduction include advanced age, female gender, osteoporosis, comminution, segmental quadrilateral plate injury, associated column fracture, Gull sign, marginal impaction, femoral head involvement, and posterior wall comminution [10]. Inability to achieve an anatomic reduction is associated with increased risk (up to 64%) of requiring conversion to THA within three years following initial ORIF [8,18-22].

Acute arthroplasty for acetabular fractures is more technically challenging than a primary hip arthroplasty and does have a higher complication rate. THA dislocation in the setting of an acetabular fracture with ORIF has been reported to be approximately 6.1% and up to 23% when performed simultaneously [2,6]. This investigation had no patients with THA dislocation following treatment with a trabecular metal cage with either a cemented bimobile component or a polyethylene liner and simultaneous ORIF. This is likely due to the added stability of large head elements afforded by the polyethylene and later dual mobility constructs. Thus, both surgical techniques can be utilized to obtain and maintain a stable construct. One prior study of acute THA with dual mobility cups used to treat acetabular fractures with concomitant ORIF had dislocation rates of 2.2% [23] and another investigation found the dislocation rate following THA for trauma is more than four times higher when using conventional bearings in comparison to dual mobility acetabular components [24].

A majority (89%, n = 41) of patients were made weight-bearing as tolerated following surgery, with all four patients who were made non-weight-bearing being in the cemented metal bimobile acetabular component group. This decision was made by the treating surgeon following intraoperative testing of pelvic stability, and the difference between groups was not statistically significant. Overall, the proportion of patients able to bear weight immediately is a much higher number than treating with acute ORIF and is paramount when treating elderly patients with the ultimate goal of mobilization. Furthermore, most patients used a walker following surgery in both groups. Six patients in the cemented bimobile group were using a wheelchair, but this difference was not statistically significant.

Periprosthetic joint infection (PJI) rates in the literature have been reported to be as high as 2-32%, which is understandable given the prolonged nature of the ORIF procedure with acute THA [25]. This investigation did not have any PJIs; however, there was one superficial infection, which was treated with oral antibiotics. Three patients in the cemented bimobile group and one patient in the polyethylene group had a sciatic nerve palsy in the common peroneal distribution and had a foot drop. Two of these patients had neuropathy prior to surgery, one had Parkinson’s, and the other was experiencing dementia. In the senior author's clinical practice, an increased number of patients with sciatic nerve palsy has been observed in the presence of pre-existing neuropathy.

Heterotopic ossification (HO) rates in the literature have previously been quoted up to 19.5%, with Brooker III and IV up to 6.8% [6]. This study had a rate of HO of 6.5% with Brooker III and IV HO. One patient was in the polyethylene group, and one patient was in the metal bimobile group. No patients required intervention for their HO.

There is a significant difference in the ages between the two groups, with those in the polyethylene component (earlier time period) having an average age of 63 and the metal bimobile group (later time period) with an average age of 73. It is possible that the longer LOSs in the metal bimobile group are a result of the older age of the patients. There were significant organizational changes during the timeframe in which the senior surgeon shifted in technique, including the home institution acquiring multiple outlying facilities. This resulted in a marked influx of referrals for geriatric falls, and we believe it is the main underlying reason for the significant differences in age between the groups.

This study has limitations due to its retrospective nature and the selection biases of the orthopedic treating surgeons deciding on the treatment plan for each patient. Additionally, this study may present a type II error due to the low number of patients.

## Conclusions

In conclusion, the treatment of acetabular fractures with a trabecular metal cage with either a cemented bimobile component or a polyethylene liner with subsequent ORIF is a comparable treatment option for elderly acetabular fractures, particularly those patients with pre-existing OA, osteoporosis, concomitant femoral head fractures, and marginal impaction. Dislocation, reoperation, neurologic deficit, and HO rates are comparable between patients treated with a cemented bimobile component and a polyethylene liner.
